# Harnessing ferroptosis to transform glioblastoma therapy and surmount treatment resistance

**DOI:** 10.1038/s41420-025-02744-x

**Published:** 2025-10-07

**Authors:** Shilpi Singh, Iteeshree Mohapatra, Debashis Barik, Haoyi Zheng, Stefan Kim, Mayur Sharma, Clark C. Chen, Gatikrushna Singh

**Affiliations:** 1 Department of Neurosurgery, University of Minnesota, Minneapolis, MN USA; 2 Department of Veterinary and Biomedical Sciences, University of Minnesota, Saint Paul, MN USA; 3 Center for Computational Natural Science and Bioinformatics, International Institute of Information Technology, Hyderabad, Telangana India; 4 Department of Neurosurgery, Warren Alpert School of Medicine, Rhode Island Hospital, Brown University, Providence, RI USA

**Keywords:** Targeted therapies, Tumour biomarkers

## Abstract

Glioblastoma remains the most aggressive and treatment-resistant brain malignancy, driven by genetic heterogeneity, metabolic plasticity, and an immunosuppressive tumor microenvironment (TME). Current therapies rely on inducing tumor cell death through DNA damage; however, glioma stem cells (GSCs) upregulate compensatory DNA repair pathways, promoting resistance and tumor recurrence. Ferroptosis, an iron-dependent form of regulated cell death driven by lipid peroxidation, offers a novel therapeutic strategy to overcome therapy resistance by exploiting glioblastoma’s metabolic vulnerabilities. Unlike conventional therapies, ferroptosis bypasses DNA repair mechanisms, making it particularly effective against therapy-resistant GSCs. It reduces tumor growth by triggering iron-catalyzed oxidative stress, disrupting lipid metabolism, and pushing glioblastoma cells beyond their oxidative threshold. However, resistance mechanisms to ferroptosis, including iron metabolism regulators (IREB2 and ferritinophagy), lipid peroxidation enzymes (ACSL4 and ALOXs), and protective pathways (cystine transporters and glutathione peroxidase 4), limit its therapeutic potential. Extracellular vesicle-mediated iron transfer further contributes to ferroptosis resistance, fostering chemoresistance and radio-resistance. Beyond direct tumor killing, ferroptosis modulates the TME by releasing damage-associated molecular patterns, inducing reactive oxygen species, stimulating CD8^+^ T-cell activation, enhancing immune checkpoint blockade efficacy, and reprogramming tumor-associated macrophages toward an anti-tumor phenotype. Ferroptosis-based strategies, including glutathione peroxidase 4 inhibitors, nanoparticle-mediated iron delivery, and RNA-based therapies, offer promising avenues for enhancing glioblastoma treatment efficacy. This review highlights ferroptosis as a promising strategy for overcoming glioblastoma resistance by integrating it with chemotherapy, radiotherapy, and immunotherapy to enhance treatment efficacy. Given the complexity of glioblastoma, personalized ferroptosis-based approaches that address tumor heterogeneity, immune interactions, and metabolic adaptations are crucial for overcoming therapy resistance. Refining ferroptosis-targeted strategies by incorporating metabolic, immune, and genetic considerations can lead to more durable and effective therapies, ultimately transforming glioblastoma treatment and improving patient outcomes.

## Facts

- Glioblastoma cells depend on iron for growth but evade ferroptosis via iron regulation, revealing a targetable metabolic vulnerability.
- Ferroptosis releases DAMPs and ROS that reprogram TAMs and activate CD8⁺ T-cells, enabling simultaneous tumor killing and immune modulation.
- DHODH, a key enzyme in pyrimidine synthesis, also drives mitochondrial ferroptosis in GPX4-deficient glioblastoma cells, offering a strategy to bypass GPX4-dependent resistance.
- Ferroptosis inducers bypass MGMT-driven DNA repair, enabling elimination of TMZ-resistant glioma stem cells and enhancing standard therapy efficacy.

## Open questions

- Can ferroptosis be selectively induced in GSCs without damaging healthy brain tissue?
- What are the dominant mechanisms of ferroptosis resistance in glioblastoma, and how can they be effectively co-targeted?
- How does ferroptosis reshape the TME, and can it synergize with immunotherapy?
- Is ferroptosis a viable strategy to overcome resistance to TMZ and radiation?
- How can tumor heterogeneity and metabolic plasticity be addressed to enable personalized ferroptosis-based therapies?

## Introduction

Glioblastoma is the most aggressive and treatment-resistant brain tumor, characterized by genetic heterogeneity, metabolic adaptability, and an immunosuppressive tumor microenvironment (TME). Despite multimodal treatment strategies, including surgical resection, radiotherapy, and temozolomide (TMZ) chemotherapy, recurrence is inevitable due to glioma stem cells (GSCs) and therapy-induced resistance mechanisms [1]. A significant contributor to treatment failure is the tumor’s ability to evade cell death by upregulating DNA repair pathways, antioxidant defenses, and metabolic reprogramming, which collectively undermine standard therapies. These challenges necessitate the exploration of alternative cell death pathways that can bypass glioblastoma’s intrinsic resistance mechanisms and provide a more effective therapeutic approach [2, 3].

Ferroptosis, an iron-dependent form of regulated cell death, has emerged as a promising avenue for glioblastoma therapy. Unlike apoptosis, ferroptosis is driven by iron-catalyzed lipid peroxidation, which leads to irreversible membrane damage and oxidative collapse [4, 5]. By exploiting glioblastoma’s metabolic vulnerabilities, ferroptosis has the potential to selectively eliminate therapy-resistant tumor cells, including GSCs, while sparing normal brain tissue. However, ferroptosis resistance mechanisms including iron sequestration, lipid repair enzymes, and antioxidant systems such as cystine transporters (xCT) and glutathione peroxidase 4 (GPX4) restrict the full therapeutic potential of ferroptosis inducers, necessitating a comprehensive strategy to overcome these adaptive barriers [6, 7].

This review aims to bridge critical knowledge gaps by elucidating the underappreciated role of ferroptosis in glioblastoma therapy resistance, emphasizing its interplay with iron metabolism, lipid peroxidation, and immune modulation. Glioblastoma cells exhibit a distinct dependence on (1) iron metabolism (ferroaddiction) yet evade ferroptosis through tight regulation of iron storage, export, and utilization [7]. (2) Lipid peroxidation, a key driver of ferroptosis, is counteracted by acyl-CoA synthetase long-chain family member 4 (ACSL4) downregulation, arachidonate lipoxygenases (ALOXs) activation, and glutathione peroxidase-4 (GPX4)-dependent lipid peroxide detoxification, allowing tumor cells to escape ferroptotic cell death [8]. (3) Ferroptosis not only induces tumor cell death but also modulates the immune landscape by releasing damage-associated molecular patterns (DAMPs) and reactive oxygen species (ROS), enhancing T-cell activation and tumor-associated macrophage (TAM) polarization toward an anti-tumor phenotype [9]. By targeting these interconnected biological processes, ferroptosis can be harnessed to sensitize glioblastoma to standard treatments and improve therapeutic efficacy.

The integration of ferroptosis inducers with chemotherapy, radiotherapy, and immunotherapy presents a novel opportunity to enhance treatment responses and overcome glioblastoma resistance mechanisms. Combining ferroptosis inducers with TMZ can circumvent O^6^-methylguanine-DNA methyltransferase (MGMT)-mediated resistance, while iron metabolism regulators can enhance radiosensitivity by increasing oxidative damage. Furthermore, ferroptosis-induced immune activation provides a compelling rationale for its combination with immune checkpoint blockade therapy, making ferroptosis a promising approach to augment programmed cell death (PD)-1/PD-L1 inhibitors in glioblastoma treatment. To fully exploit ferroptosis in glioblastoma therapy, it is crucial to target key regulators of ferroptosis resistance. GPX4, the master regulator of lipid peroxidation detoxification, is overexpressed in glioblastoma [10, 11], making it a prime therapeutic target. Ferroptosis suppressor protein 1 (FSP1) functions as an independent ferroptosis suppressor by maintaining ubiquinone (CoQ10) antioxidant activity [12], highlighting its role as a co-target alongside GPX4. Dihydroorotate dehydrogenase (DHODH), a mitochondrial enzyme involved in ferroptosis regulation [13], presents an alternative pathway for inducing ferroptotic cell death, particularly in GPX4-low expressing glioblastoma cells.

Harnessing ferroptosis in glioblastoma therapy represents a paradigm shift, offering a strategy to eliminate therapy-resistant tumor cells, reshape the TME, and enhance the effectiveness of current treatments. However, given glioblastoma’s heterogeneity, metabolic and genetic complexity, a personalized, multi-targeted approach is essential to fully integrate ferroptosis into clinical practice. This review will explore how ferroptosis-based strategies can be refined to develop durable, effective therapies that have the potential to transform glioblastoma treatment and improve patient survival.

## The biological landscape of ferroptosis in glioblastoma

**Fig. 1:**
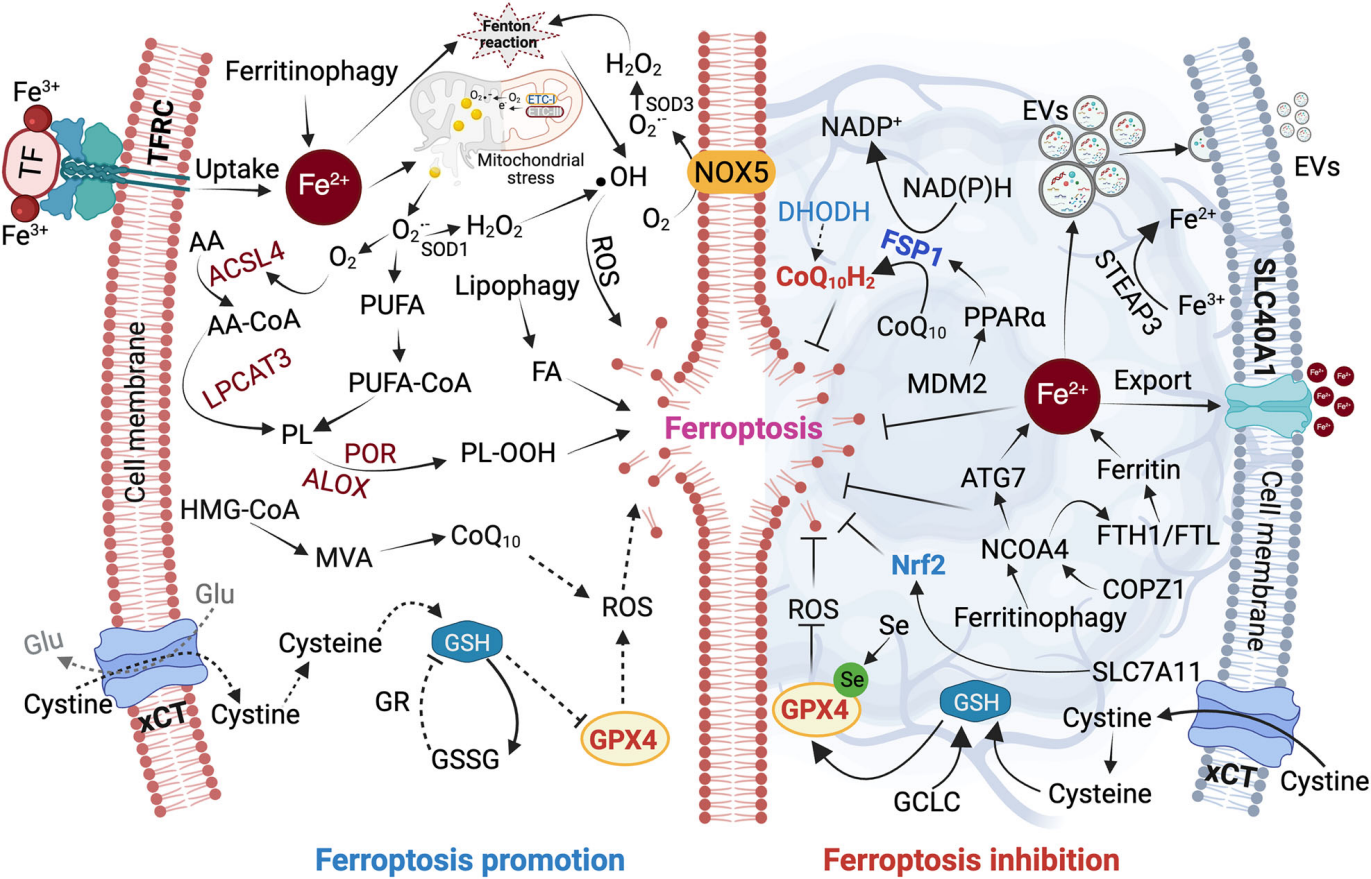
Metabolic rewiring and ferroptosis resistance in glioblastoma. This schematic illustrates the key molecular pathways regulating ferroptosis in glioblastoma. The left side represents ferroptosis-promoting mechanisms, including iron uptake via transferrin receptor (TFRC) and ferritinophagy, polyunsaturated fatty acid (PUFA) activation by ACSL4 and LPCAT3. Lipid peroxidation is further facilitated by pro-oxidant enzymes such as POR and ALOX. Concurrently, mitochondrial stress generates superoxide ions, and disruption of the inner mitochondrial membrane releases these reactive species into the cytoplasm, promoting PUFA oxidation. Additionally, NOX5-driven Fenton reactions elevate reactive oxygen species (ROS) levels, contributing to ferroptotic cell death. Depletion of intracellular glutathione (GSH), mediated by reduced cystine import via system Xc^−^ (SLC7A11/xCT), impairs GPX4 function, enabling lethal accumulation of lipid peroxides (LPO). The right-side highlights ferroptosis-inhibitory mechanisms induced by glioblastoma. These include GPX4-mediated detoxification of LPO using GSH, lipid radical scavenging via the CoQ10/FSP1 and DHODH pathways, and antioxidant gene expression regulated by Nrf2. Iron homeostasis is maintained through export (SLC40A1), ferritin-mediated sequestration (FTH1/FTL), and extracellular vesicle (EV)-mediated iron efflux, limiting labile iron pools and suppressing ROS production. Together, these networks determine cellular sensitivity to ferroptosis and highlight potential therapeutic targets in glioblastoma. The figure was created with BioRender.com.

Ferroptosis is an iron- and lipid peroxidation-dependent form of regulated cell death, emerging as a critical vulnerability in glioblastoma. ROS play a dual role in tumor progression and suppression. At moderate levels, ROS support tumorigenic processes including proliferation, angiogenesis, and therapy resistance [14–16]. However, excessive ROS accumulation can trigger ferroptosis, thereby sensitizing tumors to therapy [16]. In glioblastoma, ferroptosis is closely linked to redox homeostasis. Elevated ROS levels reduce intracellular glutathione (GSH) and suppress GPX4, a key ferroptosis inhibitor (Fig. 1). Additionally, ROS-mediated activation of p53 represses SLC7A11 transcription impairing cystine uptake via system Xc^-^ and further diminishing GPX4 activity [17, 18]

Mitochondria significantly contribute to ferroptosis via oxidative metabolism. Under cysteine deprivation or glycolytic inhibition, enhanced oxidative phosphorylation promotes LPO generation [19, 20]. Electron leakage from mitochondrial complexes I and III produces superoxide anions, which facilitate polyunsaturated fatty acid (PUFA) oxidation. This results in the accumulation of lipid hydroperoxides (LOOHs), driving ferroptotic cell death [21]. Mitochondrial lipid metabolism also generates substrates for lipid peroxidation, while ROS-induced damage to mitochondrial membranes and DNA disrupts electron transport chain (ETC) integrity, amplifying ferroptotic signals [22, 23] (Fig. 1).

Furthermore, autophagy intersects with ferroptosis through ROS-dependent activation of mTOR signaling. Under oxidative stress, mTORC1 inhibition and mTORC2-mediated AKT phosphorylation enhance autophagosome formation via Unc-51-like kinase (ULK) and PI3KC3 complex activation [24–26]. This facilitates autophagy-dependent ferroptosis, further amplifying lipid peroxidation under stress conditions.

Therapeutically, ferroptosis represents a promising strategy to overcome glioblastoma resistance. ROS-inducing agents selectively elevate oxidative stress in tumor cells, disabling antioxidant defenses such as GPX4 and system Xc⁻. This leads to enhanced chemosensitivity and preferential tumor cell elimination, offering a viable approach to target therapy-resistant GSCs while sparing normal brain tissue.

### Ferroptosis: a non-apoptotic cell death pathway

Ferroptosis offers a novel strategy to eliminate therapy-resistant glioblastoma cells. Unlike apoptosis, which is frequently bypassed in glioblastoma through mutations in p53, upregulation of anti-apoptotic proteins (BCL-2, MCL-1), or enhanced DNA repair mechanisms, ferroptosis circumvents these resistance pathways by inducing oxidative membrane damage independent of caspase activation [4, 27]. This process occurs when PUFA in membrane phospholipids undergo peroxidation due to the generation of ROS via the Fenton reaction. The inability of antioxidant systems, such as GPX4 and FSP1, to detoxify LPOs lead to irreversible membrane damage and cell death. In glioblastoma, ferroptosis presents a unique vulnerability, as tumor cells exhibit ferroaddiction, an increased dependence on iron (Fe^2+^) for metabolic functions, yet simultaneously deploy protective mechanisms to evade ferroptotic cell death (Fig. 1). Targeting these vulnerabilities by modulating iron metabolism and lipid peroxidation pathways offers a promising therapeutic avenue [28, 29].

Beyond its ability to bypass apoptotic resistance, ferroptosis presents additional advantages over other cell death pathways such as necroptosis and pyroptosis, which rely on receptor-interacting protein kinases (RIPKs) or inflammasome activation, respectively, to trigger cell death. The immunosuppressive TME of glioblastoma limits the effectiveness of these pathways, whereas ferroptosis bypasses immune modulation by directly inducing oxidative stress-mediated cell destruction. This makes ferroptosis a potent alternative in immune-evasive tumors [3]. Furthermore, glioblastoma’s dependency on lipid metabolism creates a unique therapeutic window, as lipid peroxidation regulators like ACSL4 and ALOXs can be targeted to enhance ferroptotic sensitivity [30] (Fig. 1). By exploiting lipid peroxidation and iron metabolism dysregulation, ferroptosis provides a mechanism to eliminate therapy-resistant glioblastoma cells while minimizing reliance on immune system activation.

### Iron metabolism and ferroptosis resistance in glioblastoma

Iron metabolism plays a pivotal role in ferroptosis susceptibility. Glioblastoma cells rely on ferroaddiction to drive metabolic adaptation and accelerates tumor progression. Excess Fe^2+^ in the labile iron pool (LIP) participates in the Fenton reaction, generating hydroxyl radicals (•OH) that trigger lipid peroxidation and ferroptosis. However, glioblastoma counteracts ferroptosis by tightly regulating iron uptake, storage, and export [8, 9].

Iron homeostasis is primarily modulated by iron-regulatory proteins (IRPs), ferritinophagy, and iron transport systems. Iron-responsive element-binding protein 2 (IREB2) enhances ferroptosis by increasing iron availability, while nuclear receptor coactivator 4 (NCOA4)-mediated ferritinophagy releases stored Fe^2+^, amplifying ferroptotic stress [31]. Conversely, glioblastoma cells exploit these pathways to maintain iron balance while preventing toxic accumulation. Overexpression of ferritin heavy chain 1 (FTH1) and ferritin light chain (FTL) stabilizes iron storage, limiting the free Fe^2+^ available for ferroptosis [32, 33] (Fig. 1). Additionally, glioblastoma cells enhance iron export via SLC40A1 and buffer mitochondrial iron through Fe-S cluster biosynthesis proteins, such as NFS1 and frataxin, mitigating oxidative stress [34, 35].

Glioblastoma-specific adaptations include the dysregulation of COPI coat complex subunit zeta 1 (COPZ1), which modulates the COPZ1/NCOA4/FTH1 axis to stabilize ferritin and suppress ferroptosis. COPZ1 knockdown in glioblastoma cell lines (U87MG, U251, and P3) induces ferritinophagy, upregulates NCOA4, and activates autophagy-related protein ATG7, leading to excessive Fe^2+^ release, ROS accumulation, and ferroptotic cell death [36]. Moreover, the six-transmembrane epithelial antigen of prostate (STEAP) family contributes to iron homeostasis in glioblastoma. While STEAP3 is upregulated to enhance Fe^3+^ to Fe^2+^ reduction, STEAP2 downregulation correlates with reduced ferroptosis sensitivity (Fig. 1). Gene ontology (GO) analysis links STEAP proteins to immune regulation and cell cycle control, reinforcing their role in tumor progression [37].

### Lipid peroxidation: a therapeutic vulnerability in glioblastoma

Lipid peroxidation is a central driver of ferroptosis, leading to oxidative membrane damage and cell death [38]. In glioblastoma, ferroptosis susceptibility is dictated by PUFAs integrated into phospholipids, which are prone to oxidation [39]. ACSL4 and lysophosphatidylcholine acyltransferase 3 (LPCAT3) facilitate PUFA incorporation into membrane phospholipids [40], while lipoxygenases (LOXs), such as ALOX5 and ALOX15, catalyze the formation of LOOHs, triggering ferroptosis [41] (Fig. 1). However, glioblastoma cells develop resistance by actively suppressing lipid peroxidation through adaptive metabolic alterations [42].

A key resistance mechanism is the downregulation of ACSL4, which restricts PUFA incorporation into membrane phospholipids, limiting peroxidation and ferroptotic vulnerability. ACSL4-deficient glioblastoma cells exhibit lower ferroptosis sensitivity, whereas their overexpression enhances lipid peroxidation, leading to ferroptotic cell death [43]. Additionally, glioblastoma cells upregulate GPX4, the central ferroptosis suppressor that neutralizes LPOs, preventing membrane rupture [10]. Other lipid repair mechanisms, including increased expression of ALOX15 and fatty acid desaturases (FADS2), further contribute to ferroptosis evasion by modifying phospholipid composition and reducing peroxidation-prone PUFAs [44].

MicroRNAs (miRNAs) and lipid metabolism-related genes also influence ferroptosis resistance in glioblastoma. miR-670-3p suppresses ACSL4, limiting LPO formation and reducing TMZ efficacy [45]. Similarly, miR-18a targets ALOXE3, decreasing lipid oxidation and promoting glioblastoma cell survival. Conversely, restoring ACSL4 or inhibiting miR-670-3p enhances ferroptosis sensitivity, presenting potential therapeutic strategies to improve glioblastoma treatment response [46]. Beyond miRNAs, lipid-related genes such as CYP2E1, MDM2 (murine double minute 2), and MDMX (murine double minute 4) further modulate ferroptosis susceptibility. CYP2E1 amplifies lipid peroxidation by increasing ROS production, while its downregulation correlates with poor prognosis and tumor progression [47]. MDM2 and MDMX influence ferroptosis through a p53-independent mechanism by modulating lipid metabolism via peroxisome proliferator-activated receptor-alpha (PPARα) and FSP1 that reduce CoQ10 to counteract lipid peroxidation [48].

### Cysteine transporter (xCT): a key modulator of ferroptosis resistance in glioblastoma

The cystine/glutamate antiporter system xCT (SLC7A11/SLC3A2) is a critical regulator of ferroptosis susceptibility in glioblastoma. As ferroptosis is driven by lipid peroxidation and oxidative stress, xCT sustains glioblastoma survival by maintaining intracellular cysteine levels, which serve as a precursor for GSH synthesis, a key antioxidant that mitigates ROS-induced lipid peroxidation [49–51]. The overexpression of xCT is a hallmark of glioblastoma, contributing to therapy resistance, tumor progression, and poor patient prognosis. By enabling metabolic plasticity, glioblastoma cells exploit xCT to withstand oxidative stress, nutrient deprivation, and high cell density conditions. While xCT-mediated cysteine uptake enhances GSH production and prevents ferroptotic cell death, paradoxically, excessive xCT activity can lead to oxidative stress and cytotoxicity under glucose-deprived conditions [52].

The regulation of xCT is highly dynamic in glioblastoma. Studies indicate that mTOR inactivation and lysosomal degradation of SLC7A11 at high cell density improve glioblastoma cell survival under metabolic stress [53], while epidermal growth factor (EGF) signaling paradoxically promotes ferroptosis via xCT overactivation [52]. Additionally, xCT activity extends beyond metabolic regulation to influence immune suppression in the glioblastoma TME. Excess glutamate release from xCT promotes regulatory T-cell (Treg) activation, reinforcing glioblastoma’s immunosuppressive landscape. This suggests that xCT modulation could enhance antitumor immunity while increasing ferroptosis sensitivity, positioning it as a dual-purpose therapeutic target.

Clinically, high xCT expression correlates with poor prognosis, with glioblastoma patients exhibiting shorter progression-free and overall survival when xCT is upregulated [54]. p53 negatively regulates xCT expression, and reactivation of p53 has been shown to suppress xCT activity, enhance ferroptosis sensitivity, and inhibit tumor growth [55]. Additionally, xCT supports mitochondrial biogenesis, ATP production, and ROS detoxification, helping glioblastoma cells evade chemotherapy, radiotherapy, and immunotherapy-induced ferroptosis [56–59]. However, its inhibition reduces tumor invasion, GSCs self-renewal, and progression, reinforcing its therapeutic potential.

### Glutathione peroxidase 4 (GPX4): guardian against ferroptosis in glioblastoma

The inability to eliminate LOOHs from PUFA-containing phospholipids is a defining characteristic of ferroptosis [60]. GPX4 is the central enzyme preventing ferroptosis by converting LOOHs into their non-toxic lipid alcohol forms, thereby maintaining membrane integrity and mitigating oxidative damage [61, 62]. GPX4 activity is strictly dependent on GSH, a critical antioxidant that neutralizes ROS and inhibits lipid peroxidation. When GSH levels are depleted, GPX4 becomes dysfunctional, allowing lipid peroxidation to escalate, leading to ferroptotic cell death [63]. Notably, glioblastoma cells exhibit high GPX4 expression, making them inherently resistant to ferroptosis, as GPX4 suppression correlates with increased ferroptotic sensitivity [64] (Fig. 1).

### Alternative ferroptosis regulatory pathways

Ferroptosis extends beyond GPX4-dependent mechanisms, incorporating alternative pathways that influence oxidative stress, mitochondrial integrity, and lipid metabolism. Recent findings highlight FSP1 and DHODH as critical modulators of glioblastoma ferroptosis resistance, operating independently of GPX4. Additionally, regulatory pathways such as Keap-Nrf2, Beclin-1 (BECN1), and ferritinophagy modulate ferroptosis dynamics, providing promising therapeutic targets.

#### FSP1 and DHODH: independent ferroptosis regulators in glioblastoma

FSP1, localized at the plasma membrane, counteracts ferroptosis by converting CoQ10 into ubiquinol (CoQH₂), an antioxidant that neutralizes LPOs [65]. This protective mechanism enhances glioblastoma survival by mitigating oxidative damage. Pharmacological inhibition of FSP1 disrupts CoQ10 regeneration, increasing lipid peroxidation and sensitizing glioblastoma cells to ferroptosis inducers. Conversely, DHODH, a mitochondrial enzyme involved in pyrimidine biosynthesis, operates as a ferroptosis regulator by reducing CoQ10 within mitochondria, thereby limiting lipid peroxidation [66] (Fig. 1). Inhibition of DHODH, particularly with Brequinar, has been shown to induce ferroptosis in GPX4-low-expressing glioblastoma cells and sensitize GPX4-high-expressing cells to ferroptosis inducers [67]. The dual functionality of DHODH-as both a pyrimidine synthesis enzyme and a mitochondrial antioxidant-positions it as a potential ferroptosis target in glioblastoma therapy.

#### Keap1-Nrf2 axis: a barrier to ferroptosis in glioblastoma

The Keap1-Nrf2 signaling axis is a critical regulator of the antioxidant stress response and serves as a major determinant of glioblastoma resistance to ferroptosis by enhancing cellular antioxidant defenses. Under physiological conditions, this pathway contributes to the protection of normal cells and the suppression of tumorigenesis. In response to oxidative stress, Nrf2 dissociates from Keap1 and translocate to the nucleus, where it activates antioxidant response element (ARE)-dependent genes involved in GSH synthesis and LPO detoxification [68]. Nevertheless, this protective mechanism can be co-opted during tumorigenesis; upregulation of Nrf2 has been implicated in promoting tumor proliferation as well as drug resistance. In glioblastoma, elevated Nrf2 expression correlates with poor prognosis and enhanced ferroptosis resistance, in part through the direct upregulation of SLC7A11 (Fig. 1). Emerging therapeutic strategies aimed at overcoming Nrf2-driven ferroptosis resistance include the use of inhibitors such as triptolide and brusatol, which impair antioxidant defenses and sensitize isocitrate dehydrogenase 1 (IDH1)-mutant gliomas to ferroptotic cell death [69]. Additionally, TMZ has been shown to enhance ferroptosis by inhibiting the Nrf2/Heme Oxygenase-1 (HO-1) signaling pathway, further destabilizing glioblastoma redox homeostasis [70]. For example, co-administration of TMZ with a GSH synthesis inhibitor, L-buthionine [S,R]-sulfoximine (BSO), has been shown to significantly enhance TMZ-induced DNA damage [71].

#### BECN1: a link between autophagy and ferroptosis in glioblastoma

BECN1, a core autophagy regulator, serves a dual function in glioblastoma progression and ferroptosis regulation. BECN1 directly inhibits SLC7A11, suppressing cystine uptake and increasing ferroptosis sensitivity [72]. Additionally, AMPK-mediated phosphorylation of BECN1 amplifies ferroptotic signaling, positioning it as a promising target in ferroptosis-based glioblastoma therapy [73].

#### SOCS1 and p53: tumor suppressors driving ferroptosis sensitivity

Suppressor of cytokine signaling 1 (SOCS1) enhances p53-mediated ferroptosis induction by downregulating SLC7A11, thereby reducing cystine uptake and depleting GSH levels [74]. SOCS1 overexpression also increases glioblastoma radiosensitivity, suggesting a combined ferroptosis-inducing and radiotherapy-enhancing effect. p53 plays a context-dependent role in ferroptosis regulation. In its wild-type form, p53 suppresses SLC7A11 transcription, limiting cystine uptake and sensitizing glioblastoma cells to ferroptosis [75]. However, mutant p53 can either suppress or promote ferroptosis, depending on TME conditions [76]. These findings position p53 manipulation as a strategy for ferroptosis-based glioblastoma therapy.

#### Non-coding RNAs as regulators of ferroptosis in glioblastoma

Emerging evidence underscores the role of non-coding RNAs in modulating ferroptosis sensitivity and therapy resistance in glioblastoma. miR-670-3p and miR-18a have been shown to regulate ACSL4 and ALOXE3, two key enzymes involved in ferroptosis induction respectively. miR-670-3p suppresses ACSL4, reducing ferroptosis sensitivity and contributing to TMZ- resistance [45]. Similarly, miR-18a downregulates ALOXE3 activity, impairing ferroptosis and promoting glioblastoma cell survival, suggesting that inhibiting the miR-18a/ALOXE3 axis could enhance ferroptosis and improve treatment outcomes [46]. Conversely, miR-29b enhances ferroptosis by inhibiting GPX7, increasing glioma cell sensitivity to erastin-induced ferroptosis [77]. Overexpression of long non-coding RNA, TMEM161B-AS1 drives malignant behavior and TMZ-resistance by regulating FANCD2 and CD44 via hsa-miR-27a-3p sponging [78], further highlighting the intricate role of miRNAs in ferroptosis regulation. In addition to miRNAs, LINC01564 enhances ferroptosis resistance in glioblastoma by upregulating NFE2L2 expression, which is associated with TMZ resistance [79]. Similarly, circ-TTBK2 promotes glioma proliferation and invasion while inhibiting ferroptosis through the miR-761/ITGB8 axis. Furthermore, upregulates PDGFRA via miR-3938 sponging, promoting glioblastoma progression and ferroptosis resistance, underscoring the potential of circular RNAs as novel ferroptosis modulators [80].

#### Epigenetics and tumor heterogeneity modulates ferroptosis

Epigenetic regulation and tumor heterogeneity significantly influence the susceptibility of glioblastoma cells to ferroptosis. Chromatin remodeling factors, such as HELLS, suppress ferroptosis by altering the expression of lipid metabolism genes like SCD and FADS2, which regulate monounsaturated fatty acid (MUFA) synthesis to counteract lipid peroxidation [81–83]. The hypoxia-driven regulation of HELLS by MYC and hypoxia-inducible factor 1-alpha (HIF-1α) further enhances glioblastoma’s adaptability to oxidative stress, reinforcing ferroptosis resistance [84]. Similarly, the histone demethylase KDM3B prevents ferroptosis through ATF4-dependent upregulation of SLC7A11 [85]. Additionally, epigenetic mechanisms such as H2B mono-ubiquitination promote SLC7A11 expression, linking ferroptosis regulation to the ubiquitin-proteasome system [86]. Pharmacological inhibition of BRD4 with JQ1 disrupts these ferroptosis-suppressive pathways by downregulating GPX4, SLC7A11, and SLC3A2 while promoting ferritinophagy [87], highlighting the therapeutic potential of targeting chromatin modulators to enhance ferroptosis sensitivity in glioblastoma.

Beyond epigenetic regulation, oncogenic signaling pathways contribute to ferroptosis evasion in glioblastoma. EGFR mutations, particularly EGFRvIII, drive tumor progression by modulating lipid metabolism and cystine dependency. EGFR-mutant glioblastoma cells are highly reliant on cystine uptake, making them vulnerable to ferroptosis upon cystine deprivation [7]. EGFR inhibitors such as gefitinib, erlotinib, and cetuximab have been shown to induce ferroptosis [88–90], underscoring their potential as ferroptosis-sensitizing agents. Meanwhile, IDH1-mutant gliomas exhibit distinct metabolic vulnerabilities [91], as the oncometabolite 2-hydroxyglutarate (2-HG) increases oxidative stress and enhances ferroptosis susceptibility [92]. Targeting glutaminase in IDH1-mutant gliomas disrupts GSH synthesis [93], further sensitizing these tumors to ferroptosis. Additionally, GSCs maintain ferroptosis resistance through elevated transferrin receptor (TFRC) expression, increased iron intake, and ferritin overexpression, which shields them from iron-mediated oxidative damage [94]. However, ferritin degradation serves as a potential therapeutic vulnerability, as siRNA-mediated knockdown of ferritin impairs GSC survival [95]. Combining ferroptosis inducers with autophagy modulators, such as hydroxychloroquine and TMZ, enhances ferroptotic cell death in GSCs [96], offering a promising strategy to overcome glioblastoma resistance and target the tumor’s most resilient cell population.

Overall, the diverse regulatory mechanisms governing ferroptosis in gliomas provide multiple therapeutic targets for overcoming drug resistance and enhancing the efficacy of treatments like TMZ and radiotherapy. Targeting key proteins and signaling pathways that modulate ferroptosis sensitivity, such as GPX4, FSP1, Nrf2, and others, represents a promising strategy for the development of novel therapies to combat glioblastoma.

## Tumor microenvironment and ferroptosis modulation

Ferroptosis plays a pivotal role in shaping the tumor immune microenvironment by influencing immune cell recruitment, polarization, and activation [97]. Unlike apoptosis or necrosis, ferroptotic cell death releases lipid peroxidation-derived signals and DAMPs, which modulate immune responses [98]. In the immunosuppressive glioblastoma, ferroptosis can serve as a mechanism to counteract immune evasion by reprogramming TAMs and enhancing T-cell activation. However, the interplay between ferroptosis and immune modulation is complex, as iron metabolism and oxidative stress play dual roles in both promoting and suppressing anti-tumor immunity.

### Ferroptosis and TAMs/macrophages

**Fig. 2:**
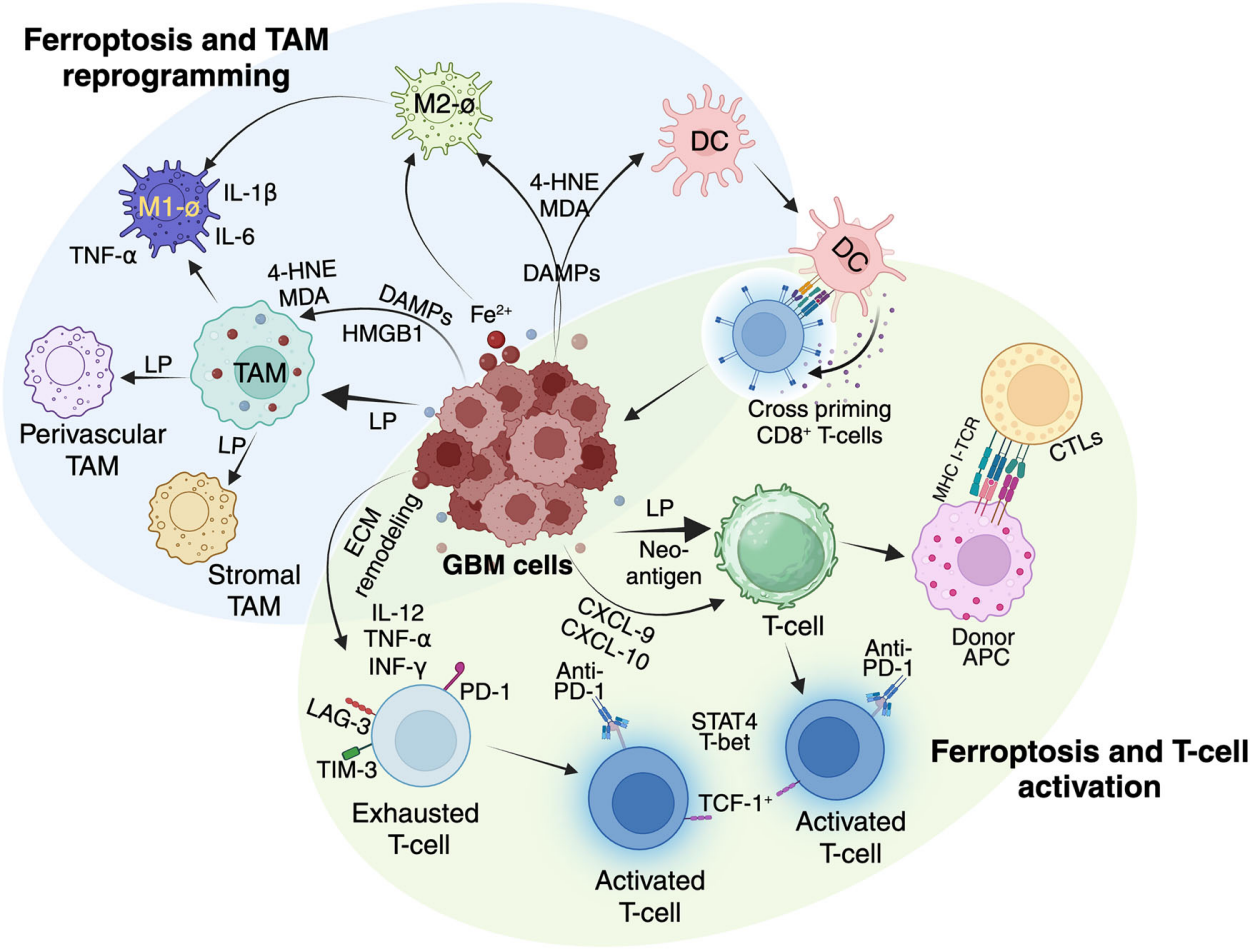
Ferroptosis-driven immunomodulation in glioblastoma. This illustration depicts how ferroptosis influences the immune landscape within the glioblastoma microenvironment. On the left, ferroptotic glioblastoma cells release damage-associated molecular patterns (DAMPs), including 4-hydroxynonenal (4-HNE), malondialdehyde (MDA), and HMGB1, which modulate tumor-associated macrophages (TAMs). Lipid peroxidation (LP) products and iron (Fe²⁺) promote TAM reprogramming, converting perivascular and stromal TAMs into pro-inflammatory M1-like macrophages that secrete TNF-α and IL-1β, while suppressing immunosuppressive M2-like phenotypes. On the right, ferroptotic glioblastoma cells also enhance dendritic cell (DC) activation and cross-priming of CD8⁺ T-cells by releasing tumor neoantigens and lipid peroxidation (LP) byproducts. These signals promote T-cell recruitment (via CXCL9/10), activation, and cytotoxic responses, including engagement of cytotoxic T lymphocytes (CTLs) and antigen-presenting cells (APCs). However, persistent antigen exposure and extracellular matrix (ECM) remodeling can also lead to T-cell exhaustion and rescue by IL-12, TNF-α and IL-1β. Together, the figure highlights the dual role of ferroptosis in modulating innate and adaptive immunity in glioblastoma. The figure was created with BioRender.com.

TAMs, which are a predominant immune component of the glioblastoma TME, are key regulators of ferroptosis-associated immune responses. These macrophages exist in a spectrum of phenotypes, ranging from pro-inflammatory M1-like TAMs, which exhibit anti-tumor functions, to immunosuppressive M2-like TAMs, which facilitate tumor progression [99]. Iron metabolism critically influences macrophage polarization, with iron overload favoring M1-like polarization, characterized by increased production of pro-inflammatory cytokines such as IL-6, TNF-α, and IL-1β. Conversely, chronic iron accumulation can promote M2-like polarization, supporting an immunosuppressive environment [100]. During ferroptosis, the release of iron and lipid peroxides triggers TAM reprogramming, shifting them toward an anti-tumor M1-like phenotype. Additionally, ferroptotic cells release high-mobility group box 1 (HMGB1), which, through the advanced glycosylation end-product receptor (AGER), enhances macrophage recruitment and inflammatory responses (Fig. 2). This immune-stimulatory effect is further amplified by the interaction of oxidized phospholipids with macrophage TLR2 receptors, increasing phagocytic activity and tumor clearance [101]. In contrast, macrophages are closely linked to iron and lipid metabolism, with M1-macrophages being more prone to ferroptosis than M2. M1-macrophages exhibit higher expression of iron storage genes (Hamp, FTH, FTL), lower ferroportin (FPN) and IRP1/2 levels, and increased ROS and inducible nitric oxide synthase (iNOS) activity. iNOS activation depletes GSH and GPX, leading to lipid peroxidation and promoting ferroptosis [102]. Targeting ferroptosis-induced macrophage reprogramming offers a promising avenue to modulate the glioblastoma immune landscape, reducing immune suppression and enhancing tumoricidal activity.

### Iron recycling and TAM metabolism

Iron metabolism plays a crucial role in shaping the functional state of TAMs, influencing their capacity to either suppress or promote anti-tumor immunity. A subset of iron-loaded, pro-inflammatory TAMs (iTAMs) has been identified in hemorrhagic tumor regions, where their presence correlates with reduced tumor burden in non-small cell lung cancer. These iron-laden TAMs, when exposed to hemolytic red blood cells or iron oxide nanoparticles, undergo repolarization into tumoricidal macrophages, a phenomenon that has demonstrated therapeutic efficacy in vivo by significantly reducing tumor size [103]. This suggests that iron supplementation, particularly through nanoparticle-mediated delivery, can serve as a strategy to reprogram TAMs into an anti-cancer phenotype.

Distinct TAM subsets display variable responses to iron metabolism, with iTAMs exhibiting high iron content and an enrichment of heme and iron metabolism-related genes. These macrophages, categorized into perivascular (pviTAM) and stromal (stiTAM) subtypes, promote angiogenesis and immunosuppression, facilitating tumor progression. The transcriptional regulator Bach1 represses the iTAM program, while heme exposure induces its activation, highlighting a potential target for modulating TAM function [103]. In hepatocellular carcinoma, tumor cells outcompete macrophages for iron by upregulating TFRC, shifting TAMs toward an M2-like phenotype that enhances immune suppression (Fig. 2). Elevated TFRC expression is associated with increased M2-macrophage infiltration and worse clinical outcomes, underscoring the role of iron competition as a key mechanism in tumor immune evasion [104]. Given these findings, targeting iron metabolism in TAMs offers a promising approach to modulate macrophage polarization and enhance ferroptosis-driven anti-tumor immunity.

### Ferroptosis-mediated activation and reinvigoration of CD8^+^ T-cells

In glioblastoma, a lipid-rich, hypoxic, and immunosuppressive TME fosters CD8⁺ T-cell exhaustion, marked by high PD-1, TIM-3, and LAG-3 expression [105]. Ferroptosis plays a multifaceted role in remodeling the immune microenvironment, primarily by enhancing CD8⁺ T-cell-mediated antitumor immunity. Early-stage ferroptotic tumor cells release HMGB1, ATP, and calreticulin, which drive dendritic cell (DC) maturation and antigen presentation, promoting robust CD8⁺ T-cell priming and infiltration [106–109]. This immunostimulatory effect is temporally regulated, with early ferroptotic cells exhibiting maximal immune-activating potential. Concurrently, ferroptosis reprograms TAMs, shifting them from immunosuppressive M2- to pro-inflammatory M1-phenotypes via iron overload, ROS accumulation, and modulation of GPX4, NF-κB, xCT, and TLR2 signaling [110, 111]. These reprogrammed M1-macrophages secrete IL-12, TNF-α, and IFN-γ, further amplifying CD8⁺ T-cell cytotoxic function and enhancing the efficacy of immune checkpoint blockade [112]. In addition, myeloid-derived suppressor cells (MDSCs) and tumor-infiltrating neutrophils are also susceptible to ferroptosis. Their targeted depletion via ferroptotic pathways diminishes immunosuppression, facilitates T-cell activity, and improves checkpoint blockade responses [113–115]. Regulatory T-cells (Tregs), which resist ferroptosis by upregulating GPX4, become vulnerable upon GPX4 inhibition, resulting in reduced immune suppression and improved CD8⁺ T-cell activity [116].

Ferroptosis also remodels redox balance in the TME, depleting immunosuppressive lipids and restoring mitochondrial metabolism in T-cells, thereby enhancing oxidative phosphorylation and effector function [117]. Ferroptosis-driven inflammation upregulates IL-12 and type I interferons, activating transcription factors like STAT4 and T-bet that reprogram exhausted CD8⁺ T-cells epigenetically [117]. In parallel, ferroptosis-induced extracellular matrix remodeling facilitates T-cell infiltration, creating a more favorable immunotherapeutic landscape. Furthermore, ferroptotic ROS and lipid peroxides affect non-immune stromal elements disrupting astrocyte metabolism, impairing glutamate homeostasis, and weakening the blood-brain barrier (BBB), thereby enhancing immune cell trafficking and reshaping the TME.

Ferroptosis-inducing nanotherapeutics including polymer-based nanoparticles, metal ion complexes, and combination regimens with radiotherapy or photodynamic therapy further enhance tumor clearance by inducing oxidative stress, lipid peroxidation, and glutathione depletion. These agents not only kill tumor cells directly but also remodel the TME by promoting M1-macrophage polarization, suppressing immunosuppressive mediators (PD-L1, IDO-1, TGF-β), and fostering durable CD8⁺ T-cell responses [118–123]. Ferroptosis inducers combined with PD-1 blockade expand the TCF1⁺ stem-like progenitor CD8⁺ T-cell population, which is critical for durable antitumor responses [124]. Notably, nanocarriers such as gold nanoparticles loaded with miR-21-3p synergize with immune checkpoint inhibitors by disrupting redox balance and sensitizing tumors to ferroptosis [125]. Similarly, inhibition of stearoyl-CoA desaturase 1 (SCD1), radiation, and nanopolymer-triggered ferroptosis activate immunogenic pathways and enhance T-cell infiltration, offering potent combinatorial strategies for glioblastoma immunotherapy [126, 127].

Collectively, these insights underscore ferroptosis as a potent immunologic catalyst capable of reversing T-cell exhaustion and enhancing immunotherapeutic efficacy. Targeting ferroptosis within integrative treatment frameworks offers a promising strategy to overcome glioblastoma’s immunosuppressive barriers and reinvigorate antitumor immunity.

### Immune-ferroptosis interplay

The intersection between ferroptosis and antitumor immunity represents a pivotal yet underexplored frontier in glioblastoma therapeutics. Mounting clinical evidence reveals that ferroptosis induction exerts profound immunomodulatory effects on the TME, with ferroptotic glioblastoma cells releasing DAMPs that increase DC activation by 4.7-fold and enhance T-cell priming efficiency by 3.2-fold compared to apoptotic cells [128]. These immunogenic properties are particularly significant in glioblastoma, where ferroptosis-related gene signatures demonstrate strong predictive value for response to PD-1 blockade in recurrent tumors, suggesting an intrinsic synergy between ferroptosis and immune checkpoint inhibition [129]. The ability of ferroptosis to counteract glioblastoma’s immunosuppressive TME is underscored by single-cell RNA sequencing data showing a 2.8-fold increase in tumor-infiltrating CD8^+^ T-cells coupled with a 72% phenotypic shift of TAMs following ferroptosis induction [130, 131]. This immune remodeling translates to substantial therapeutic benefits, with combination approaches (TMZ+anti-PD-1) achieving complete responses in 38% of preclinical models compared to 8% with immunotherapy alone [132]. Importantly, ferroptosis appears to preferentially target immunosuppressive myeloid populations, reducing granulocytic MDSCs by 3.4-fold while sparing antitumor immune effectors [133]. However, emerging evidence reveal temporal complexities in this interplaywhile acute ferroptosis stimulates antitumor immunity, chronic induction may paradoxically promote T-cell exhaustion by PD-1/TIM-3 co-expression increasing from 12% to 58% over six months of treatment [134]. This dichotomy highlights the need for optimized treatment schedules, where intermittent ferroptosis induction preserves T-cell function while maintaining tumoricidal efficacy.

Adding further complexity, the dual susceptibility of immune cells, particularly NK cells, B cells, TAMs and CD8⁺ T-cells to ferroptosis represents a critical but underexplored determinant of glioblastoma progression and therapy resistance. In glioblastoma microenvironment, chronic oxidative stress drives mitochondrial dysfunction, rendering immune effector cells highly prone to ferroptotic cell death. This limits the durability of immune responses and compromises the efficacy of immunotherapies.

NK cells exhibited impaired proliferation and cytotoxic function under lipid peroxidation stress due to disrupted glucose metabolism [135]. Among B cells, B1 and marginal zone subsets, characterized by high CD36 expression are more susceptible to ferroptosis and rely on GPX4 for survival and antibody production, whereas follicular B cells are relatively ferroptosis-resistant due to lower intracellular fatty acid levels [136]. Tregs demonstrate increased resistant compared to CD8⁺ T-cells, attributed to their secretion of thioredoxin-1 and elevated GPX4 expression. However, this protection is compromised upon activation, leading to IL-1β secretion and downstream activation of Th17 cells, DCs, and CD8⁺ T cells, ultimately amplifying antitumor immunity [116, 137]. CD8⁺ T cells are sensitive to ferroptosis during their development and activation. These cells depend on glucose, glutamine, and cysteine to fuel mitochondrial metabolism and synthesize GSH. Although early activation is typically preserved, proliferating CD8⁺ T-cells become ferroptosis-prone when GSH synthesis is impaired, leading to mitochondrial dysfunction and compromised effector function [138].

Within TME, high levels of lipids and cholesterol upregulate CD36 expression in CD8⁺ T-cells, facilitating the uptake of fatty acids and oxidized LDL. This results in endoplasmic reticulum stress, lipid peroxidation, and ferroptosis, subsequently reducing TNF-α and IFN-γ production and increasing PD-1 expression [139]. Furthermore, uptake of arachidonic acid (AA) and ox-LDL further drives ferroptosis through p38 MAPK signaling, which can be partially reversed by GPX4 overexpression [139–141]. Similarly, prostaglandin E2 (PGE2) disrupts IL-2 signaling, leading to oxidative stress and mTOR-PGC1α axis inhibition [142]. Tumor cells also outcompete T-cells for cystine by overexpressing SLC7A11, thereby reducing GSH levels and promoting ferroptotic cell death. Moreover, upregulation of SLC2A3 in tumor-infiltrating CD8⁺ T-cells correlate with metabolic stress and ferroptosis, whereas GCLC overexpression restores glutamate balance and reinvigorates T-cell function [138, 143]. Intrinsically, PLPP1 deficiency, regulated by PD-1 signaling, increases CD8⁺ T-cell sensitivity to ferroptosis induced by unsaturated fatty acid accumulation [144]. While GPX4, FSP1 overexpression, or ACSL4 deletion can protect T-cells, complete loss of ACSL4 may paradoxically dampen antitumor responses [145]. Therapeutically, blocking the adenosine A2A receptor (A2AR) in combination with lipophilic antioxidants like liproxstatin-1 protects CD8⁺ T-cells from ferroptosis and enhances the efficacy of adoptive cell therapy [146]. These insights reveal a complex interplay of metabolic, signaling, and microenvironmental factors regulating CD8⁺ T-cell ferroptosis, highlighting a spectrum of actionable targets for immunotherapeutic optimization in glioblastoma.

The immune-ferroptosis axis thus represents a double-edged sword in glioblastoma therapy. While capable of converting immunologically “cold” tumors and enhancing checkpoint blockade efficacy, it requires precise modulation to avoid immunosuppressive counteradaptations [147]. Future research must address critical knowledge gaps regarding optimal combination timing, patient stratification biomarkers, and strategies to sustain immunogenic effects while mitigating immune cell ferroptosis. As clinical validation progresses, this emerging paradigm promises to break the immunosuppressive barriers that have long limited glioblastoma immunotherapy success.

## Significance of ferroptosis in glioblastoma

Ferroptosis holds uniquely actionable relevance in glioblastoma due to its intersection with glioblastoma-specific metabolic dependencies, therapy resistance, and immunologic constraints that distinguish it starkly from its role in other tumor types. Unlike hepatocellular carcinoma, ferroptosis is largely governed by system Xc⁻ heterodimeric transporter (SLC7A11 and SLC3A2) inhibition and basal ROS stress [148], glioblastoma exhibits a GPX4-dependent survival phenotype, particularly in GSCs. In patient-derived glioblastoma models, GPX4 inhibition leads to a rapid and selective induction of ferroptosis in CD133^+^ GSCs, whereas differentiated non-GSCs remain comparatively resistant [149]. This hierarchical ferroptosis sensitivity within glioblastoma is not observed in KRAS-mutant pancreatic or lung cancers, where ferroptosis sensitivity tends to be more uniform and reliant on SLC7A11 suppression or RAS-driven oxidative stress [134]. Moreover, iron metabolism in glioblastoma is uniquely reprogrammed to facilitate ferroptotic vulnerability. Glioblastoma cells and GSCs upregulate TFRC while simultaneously downregulating FTH1, leading to elevated labile iron pools. In contrast, breast and colorectal cancer cells generally require exogenous iron supplementation to sensitize cells to ferroptosis inducers such as erastin or RSL3 [150]. This intrinsic iron priming in glioblastoma provides a basal susceptibility that can be exploited with significantly lower doses of ferroptosis inducers, reducing systemic toxicity risk. Hypoxic peri-necrotic regions in glioblastoma further sensitize cells to ferroptosis through HIF-1α-mediated repression of lipid desaturases such as SCD1, increasing the abundance of PUFAs, ideal substrates for ACSL4-dependent lipid peroxidation. In contrast, renal cell carcinoma [151] or prostate cancer [152], HIF-1α tends to suppress ferroptosis via upregulation of lipid antioxidants such as SLC7A11 or FSP1.

Furthermore, glioblastoma’s immune microenvironment uniquely modifies ferroptosis outcomes. While ferroptotic cancer cells in melanoma models release DAMPs that stimulate DC activation and enhance checkpoint blockade response, glioblastoma’s immune-restricted microenvironment complicates this process. However, ferroptosis in glioblastoma has been shown to reprogram TAMs toward an M1-like phenotype via lipid peroxidation byproducts, which is less evident in extracranial tumors with robust immune infiltration [3]. For instance, 4-HNE and oxidized phosphatidylethanolamines derived from ferroptotic glioblastoma cells upregulate IL-12 and TNF-α in infiltrating macrophages, a feature not observed in hepatoma or colorectal models [153].

Therapeutically, glioblastoma also exhibits non-redundant resistance mechanisms. Although Nrf2-mediated antioxidant signaling confers ferroptosis resistance in several cancers, glioblastoma often exhibit constitutive Keap1-Nrf2 activation, which simultaneously supports both redox buffering and DNA repair. This dual regulation does not occur in lung adenocarcinoma, where Keap1 mutations primarily drive ROS resistance. In glioblastoma, Nrf2 promotes MGMT transcription and ferroptosis resistance concurrently, creating a unique axis of redox and DNA repair crosstalk [154]. Additionally, ferroptosis serves a compensatory death pathway in TMZ-resistant glioblastoma. These resistant cells upregulate lipid ROS-detoxifying enzymes such as GPX4 and FSP1, and knockdown of GPX4 in these resistant cells re-sensitizes them to both ferroptosis and TMZ [155], highlighting a glioblastoma-specific therapeutic vulnerability that emerges from alkylating agent adaptation.

## Unique glioblastoma adaptations that evade ferroptosis

Glioblastoma exhibits remarkable metabolic flexibility, allowing it to evade ferroptosis by dynamically adjusting its nutrient utilization, redox balance, and iron homeostasis. Unlike normal cells, which rely primarily on oxidative phosphorylation or glycolysis, glioblastoma cells adapt to metabolic stress by shifting between these pathways to maintain energy production and cellular integrity [156, 157]. This adaptability extends to lipid metabolism, where glioblastoma cells preferentially modulate PUFA composition and upregulate lipid repair mechanisms, such as GPX4 and ferroptosis suppressor FSP1, to prevent lethal lipid peroxidation [56]. Additionally, glioblastoma cells exploit xCT activity to sustain GSH production, bolstering their antioxidant defenses [58]. By fine-tuning these metabolic pathways, glioblastoma cells maintain redox homeostasis and suppress ferroptosis, making them highly resistant to oxidative stress-induced cell death.

GSCs further reinforce ferroptosis resistance through their stem-like properties, which enhance iron sequestration and lipid peroxidation suppression. GSCs possess a higher expression of iron-regulatory proteins such as FTH1 and TFRC, allowing them to tightly regulate iron availability while minimizing the toxic effects of free iron-induced ROS [158]. This iron storage capacity, coupled with enhanced mitochondrial activity and metabolic plasticity, enables GSCs to withstand oxidative stress that would otherwise trigger ferroptosis [156]. Moreover, GSCs exhibit lower ACSL4 expression, reducing the incorporation of oxidizable PUFAs into membrane phospholipids, thereby limiting ferroptosis susceptibility [40]. The hypoxic TME further compounds ferroptosis resistance by stabilizing HIF-1α, which reprograms lipid metabolism and enhances antioxidant defenses. Hypoxia-driven metabolic shifts reduce intracellular iron availability through increased lactate production, limiting Fenton reaction-mediated lipid peroxidation. Additionally, hypoxia suppresses ferroptotic stress by promoting Nrf2 activation, which enhances GSH biosynthesis and upregulates lipid peroxidation detoxification pathways [159]. These adaptations collectively create a ferroptosis-resistant niche within the glioblastoma tumor, underscoring the necessity of targeting metabolic vulnerabilities to sensitize glioblastoma cells to ferroptotic cell death.

## Harnessing ferroptosis to enhance glioblastoma therapy

Overcoming therapeutic resistance is critical, and ferroptosis has emerged as a promising strategy to augment existing treatments. Mounting evidence demonstrates that ferroptosis induction can synergize with chemotherapy, radiotherapy, and immunotherapy by targeting unique vulnerabilities in glioblastoma cells while bypassing key resistance mechanisms. By targeting the unique metabolic vulnerabilities of glioblastoma cells, ferroptosis induction offers a multifaceted approach to enhance chemotherapy, radiotherapy, and immunotherapy while bypassing key resistance mechanisms.

### Ferroptosis and chemotherapy

The clinical utility of TMZ in glioblastoma treatment is significantly constrained by MGMT-mediated DNA repair mechanisms. Emerging evidence indicates that ferroptosis induction represents a promising strategy to circumvent this resistance pathway. Unlike conventional chemotherapy that targets DNA integrity, ferroptosis operates through iron-dependent lipid peroxidation, providing an orthogonal approach to tumor cell elimination [160].

The xCT (SLC7A11) serves as a critical regulator of ferroptosis sensitivity. Studies demonstrate that elevated xCT expression strongly correlates with TMZ-resistance in recurrent glioblastoma patients, with multivariate analysis revealing a hazard ratio of 2.3 for treatment failure [58]. This relationship is mechanistically explained by xCT’s role in maintaining intracellular GSH levels, where its inhibition leads to catastrophic lipid peroxidation. Similarly, targeting GPX4, the central enzymatic defense against ferroptosis, has shown remarkable synergy with TMZ. Preclinical studies indicate that GPX4 inhibition enhances TMZ cytotoxicity by 78% in MGMT-positive patient-derived xenografts, independent of MGMT promoter methylation status [161].

**Fig. 3:**
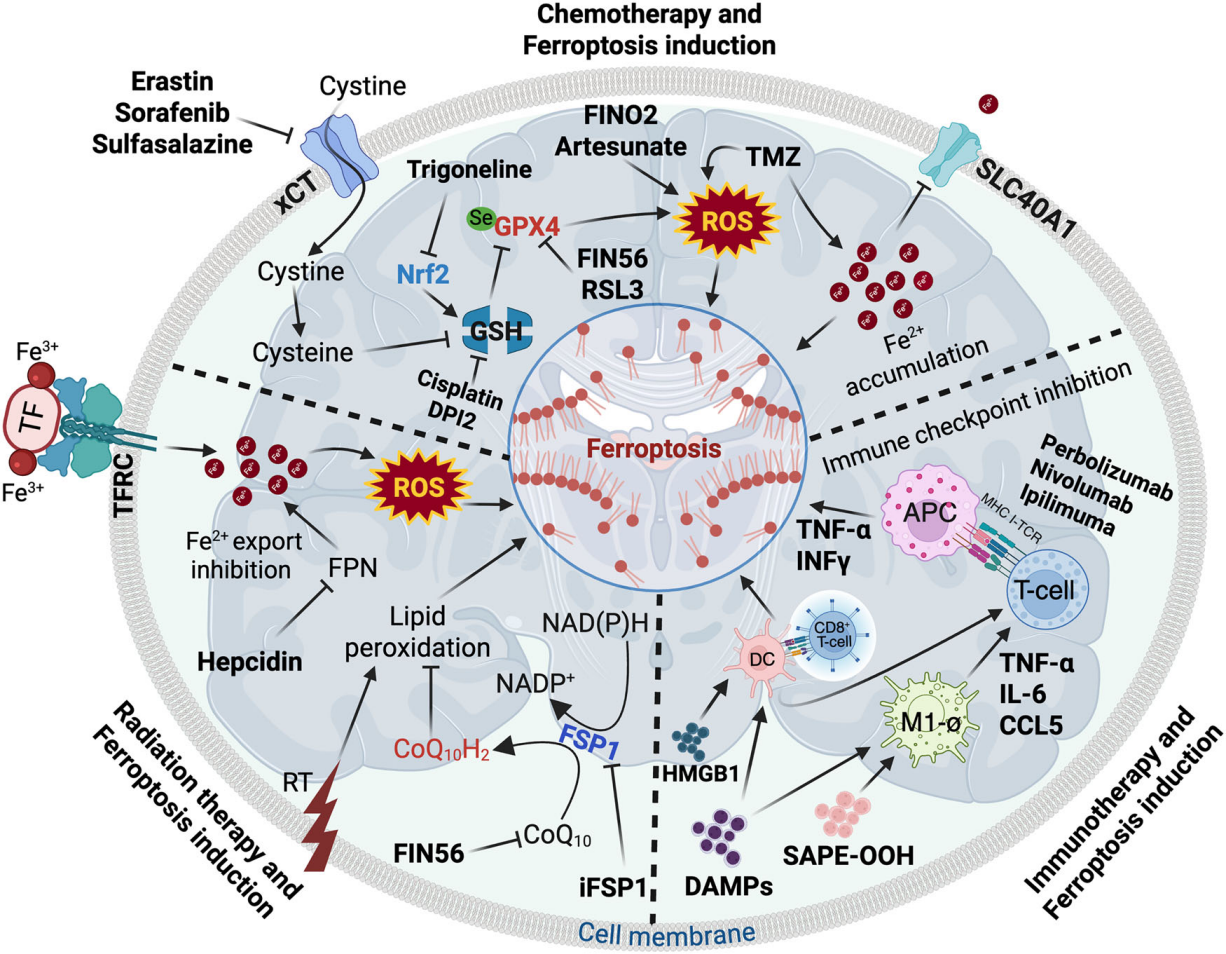
Synergistic strategies for inducing ferroptosis in glioblastoma. This schematic illustrates the integration of ferroptosis induction into multimodal glioblastoma therapies. Ferroptosis is triggered by increased intracellular iron (Fe^2^⁺) via transferrin receptor (TFRC)-mediated uptake, reduced export through ferroportin (FPN) inhibition by hepcidin, and accumulation induced by temozolomide (TMZ). Erastin and other ROS inducers disrupt cystine uptake through xCT, leading to glutathione (GSH) depletion and GPX4 inhibition, which collectively elevate reactive oxygen species (ROS) and drive lipid peroxidation. FIN56 further promotes ferroptosis via enhanced lipid peroxidation. Radiation contributes by increasing ROS levels and ferroptotic signaling. Concurrently, ferroptosis releases immunogenic damage-associated molecular patterns (DAMPs), enhancing dendritic cell (DC) activation and CD8⁺ T-cell priming. This immunogenic cell death can synergize with immune checkpoint inhibition to potentiate antitumor immunity. Together, this figure highlights the therapeutic potential of combining ferroptosis induction with chemo-, radio-, and immunotherapy to overcome glioblastoma resistance. The figure was created with BioRender.com.

These findings are supported by survival analyses demonstrating that combinatorial approaches using TMZ with ferroptosis inducers (erastin, sorafenib) extend median survival by 40–60% compared to TMZ monotherapy in orthotopic models [162]. Importantly, this therapeutic synergy is particularly pronounced in treatment-resistant tumors, suggesting ferroptosis induction may overcome both intrinsic and acquired chemoresistance mechanisms in glioblastoma (Fig. 3).

### Ferroptosis and radiotherapy

Radiotherapy resistance in glioblastoma presents a significant clinical challenge, largely attributable to robust antioxidant defenses and efficient DNA damage repair systems. Ferroptosis induction offers a novel radio-sensitization strategy by exploiting iron-mediated oxidative stress pathways. Central to this approach is the manipulation of iron metabolism regulators that control cellular redox homeostasis. TFRC, the primary mediator of iron uptake, demonstrates strong correlation with radiation response in glioblastoma [163]. Conversely, FPN, responsible for iron export, shows an inverse relationship with radiosensitivity, where its genetic knockdown enhances radiation-induced cell death by 65% in vitro [164]. These observations have been translated into therapeutic approaches using iron oxide nanoparticles, which substantially increase intracellular labile iron pools and amplify radiation-induced lipid peroxidation by 3.2-fold [165]. Clinical relevance is further supported by combination studies demonstrating that pharmacological ferroptosis inducers (FIN56) synergize with radiotherapy to reduce tumor volume by 89% compared to 52% with radiation alone in orthotopic models [164, 166]. Transcriptomic analyses of radiation-resistant glioblastoma cells reveal significant downregulation of ferroptosis-related genes, providing future molecular targets for this therapeutic approach [167]. These findings collectively establish iron metabolism modulation as a viable strategy to overcome radio-resistance in glioblastoma (Fig. 3).

### Ferroptosis and immunotherapy

The immunosuppressive TME of glioblastoma presents a formidable barrier to immunotherapy success. Ferroptosis induction has emerged as a powerful strategy to convert immunologically “cold” tumors into “hot” microenvironments through the generation of immunogenic DAMPs. Mechanistic studies reveal that ferroptotic cells release substantial quantities of HMGB1 and ATP, resulting in a 4.7-fold increase in DC activation and 3.2-fold enhancement in T-cell priming efficiency [168]. These immunostimulatory effects are clinically relevant, as ferroptosis-related gene signatures demonstrate strong predictive value for response to PD-1 blockade in recurrent glioblastoma [169]. Ferroptosis induction drives significant immunologic remodeling by increasing CD8^+^ T-cell infiltration and reduction in Treg populations in the TME [127]. Studies further demonstrate that ferroptosis inducers effectively repolarize TAMs from an M2- to M1-phenotype [102] (Fig. 3).

The strategic induction of ferroptosis addresses multiple therapeutic challenges in glioblastoma management, offering solutions to chemotherapy resistance, radio-resistance, and immunotherapy failure. The integration of ferroptosis-targeting approaches with standard therapies represents a paradigm shift in glioblastoma treatment, supported by robust preclinical evidence and early clinical data. With several ferroptosis-modulating agents currently in Phase I/II trials for glioblastoma (NCT04205357), this therapeutic strategy holds significant promise for improving outcomes in this devastating disease [170]. Future research should focus on optimizing combination regimens and developing reliable biomarkers to guide clinical implementation.

## Novel therapeutic targets to sensitize glioblastoma to ferroptosis-based treatments

GPX4 serves as the central enzymatic defense against ferroptosis by neutralizing LPOs, making it a critical therapeutic target in glioblastoma. The essential role of GPX4 is underscored by findings that its genetic ablation induces ferroptosis across multiple cancer types, including glioblastoma [171]. Clinical data reveal that GPX4 expression is elevated in therapy-resistant glioblastoma, with high GPX4 levels correlating with poor patient survival [172]. GPX4 inhibition with RSL3 demonstrates potent anti-tumor effects, reducing glioblastoma cell viability. Importantly, GPX4 inhibitors show remarkable synergy with standard therapies, enhancing TMZ cytotoxicity [173]. These findings position GPX4 as a pivotal target for overcoming glioblastoma treatment resistance.

FSP1 has emerged as a parallel ferroptosis resistance pathway independent of GPX4, functioning through the regeneration of reduced CoQ10. FSP1 expression is significantly upregulated in recurrent glioblastoma specimens (2.5-fold) compared to primary tumors [172], suggesting its role in therapeutic resistance. Mechanistically, FSP1 maintains the antioxidant capacity of the CoQ10 system, with its overexpression conferring resistance to GPX4 inhibition in 67% of glioblastoma cell lines [174]. Dual targeting of FSP1 and GPX4 demonstrates synergistic effects, increasing ferroptosis sensitivity by 4.8-fold compared to single-agent approaches [174]. These findings highlight FSP1 as a compelling secondary target for comprehensive ferroptosis induction in glioblastoma.

DHODH represents a mitochondrial vulnerability in glioblastoma, particularly those with low GPX4 expression. DHODH maintains mitochondrial redox homeostasis by regulating CoQ10 reduction, with its inhibition leading to profound lipid peroxidation. Notably, DHODH expression inversely correlates with ferroptosis sensitivity in glioblastoma, with DHODH-high tumors showing 3.1-fold greater resistance to ferroptosis inducers [175]. Pharmacological DHODH inhibition induces ferroptosis in 82% of GPX4-low expressing glioblastoma cells, while sparing normal astrocytes [176]. These findings establish DHODH as a promising mitochondrial target for ferroptosis induction, particularly in GPX4-resistant glioblastoma.

The identification of GPX4, FSP1, and DHODH as key regulators of ferroptosis resistance provides a comprehensive therapeutic framework for glioblastoma treatment. These targets collectively address the multiple layers of antioxidant defense in glioblastoma cells, offering synergistic opportunities for combination therapies. GPX4 inhibitors and other ferroptosis modulators are currently in clinical trials for various diseases (Table 1), while DHODH inhibitors demonstrating strong preclinical efficacy, represent an emerging frontier for future ferroptosis-based glioblastoma therapy. Future research should focus on novel biomarker development to identify patients most likely to benefit from these targeted strategies.

**Table 1:** Ferroptosis-targeted therapeutics in clinical trials.

Drugs	Targets	Ferroptosis	Trial No	Phase	Disease condition	Status
Altretamine	GPX4	Inducer	NCT00002936	I	Lymphoma and Sarcoma	Completed
Withaferin A			NCT00689195	I/II	Advanced osteosarcoma	Recruiting
			NCT05610735	I/II	Recurrent ovarian cancer	Recruiting
Gemcitabine			NCT06015659	II	Pancreatic cancer	Recruiting
			NCT05147272	I	Advanced solid tumor	Active, not recruiting
Neratinib	Iron activator	Inducer	NCT03377387	I/II	Metastatic HER2-positive breast cancer	Active, not recruiting
			NCT04366713	II	HER2 amplified breast cancer	Completed
Atorvastatin	HMGCR		NCT00816244	II	Postmenopausal breast cancer	Completed
Fluvastatin			NCT00416403	II	Ductal carcinoma, Stage-I breast cancer	Completed
Simvastatin			NCT03454529	II	Stage-I/II breast cancer	Completed
Cisplatin	GSH depletion	Inducer	NCT04574960	III	Upper tract invasive cancer	Recruiting
Buthionine Sulfoximine			NCT00002730	I	Neuroblastoma	Completed
			NCT00005835	I	Resistant or recurrent high-risk neuroblastoma	Completed
Acetaminophen			NCT01783236	IV	Lung tumor	Completed
Sorafenib	Cysteine transporter		NCT02559778	II	Advanced neuroblastoma	Recruiting
			NCT00449033	III	Stage-IV non-small cell lung cancer	Completed
			NCT01371981	III	Newly diagnosed acute myeloid leukemia	Active, not recruiting
			NCT01203787	IV	Hepatocellular carcinoma	Completed
			NCT00064350	II	Refractory non-small cell lung cancer	Completed
			NCT03247088	I/II	Recurrent or refractory acute myeloid leukemia	Active, not recruiting
			NCT02069145	I	Hepatocellular cancer	Completed
Sulfasalazine			NCT03847311	II	Breast cancer	Completed
			NCT04205357	I	Recurrent glioblastoma	Completed
			NCT01577966	NA	Glioma	Completed
Temozolomide			NCT01781403	I	Rectal cancer	Completed
			NCT04091503	I	Glioblastoma	Completed
	DNMT		NCT00022711	II	Relapsed or progressive small cell lung cancer	Completed
			NCT05128734	II	Triple negative breast cancer	Not yet recruiting
Leflunomide	DHODH	Inducer	NCT06454383	I	Advanced unresectable pancreatic cancer	Recruiting
			NCT05937191	I/II	Idiopathic pulmonary hemosiderosis	Recruiting
			NCT00003775	II	Anaplastic astrocytoma	Completed
			NCT06540937	II	MEN-1 neuroendocrine tumor	Recruiting
			NCT05007678	III	COVID-19	Completed
Brequinar			NCT04575038	II	COVID-19	Completed
Edaravone	Radical-trapping antioxidants	Inducer	NCT00415519	III	Amyotrophic lateral sclerosis	Completed
			NCT00200356	IV	Acute ischemic stroke	Completed
			NCT00265239	IV	Acute myocardial infarction	Completed
			NCT01865201	II	Temporal lobe necrosis	Completed
			NCT02430350	III	Acute ischemic stroke	Completed
Promethazine			NCT04805073	IV	Pruritus	Completed
Menaquinone-4			NCT00548509	IV	Bone turnover	Completed
			NCT00960973	IV	Glucose metabolism	Completed
Artesunate	ROS, NCOA4 and FTH1		NCT02633098	II	Stage-II/ III colorectal cancer	Recruiting
			NCT00764036	I	Metastatic breast cancer	Completed
Rosiglitazone	ACSL4	Inhibitor	NCT04114136	II	Solid tumor malignancies	Recruiting
			NCT00309309	II	Kidney transplant	Completed
			NCT00492700	II	Non-alcoholic steatohepatitis	Completed
			NCT00688207	I	Mild Alzheimer’s disease	Completed
			NCT00004180	II	Liposarcoma	Completed
			NCT00367744	II	HIV-1	Completed
			NCT00065065	II	Ulcerative colitis	Completed
			NCT00182052	III	Androgen dependent prostate cancer	Completed
Zileuton	ALOX5		NCT01136941	I	Sickle cell disease	Completed
			NCT00070486	II	Advanced non-small cell lung cancer	Completed
			NCT00056004	II	Bronchial dysplasia	Completed
			NCT02348203	II	Biomarker expression in nasal tissue	Completed
			NCT02348203	II	Nasal tissue of current smokers	Completed
Terameprocol	Pan LOXs		NCT02575794	I	High-grade glioma	Completed
Tetra-O-Methyl Nordihydroguaiaretic Acid			NCT00404248	I/II	Recurrent high-grade glioma	Completed
DXZ	Iron chelator		NCT03589729	II	Blood cancers	Recruiting
Deferasirox			NCT03387475	II	Low-risk MDS resistant or relapsing after ESA agents	Completed
Deferiprone			NCT05604131	II	Acute myocardial infarction	Recruiting
Deferoxamine			NCT00800761	IV	Cardiac disease in thalassemia	Completed
			NCT00777140	II	Middle cerebral artery occlusion	Completed
			NCT04633889	II	Cardiac surgery-associated acute kidney injury	Completed
			NCT04566991	II	Aneurysmal subarachnoid hemorrhage	Recruiting
Mifepristone	GSH-synthesis	Inhibitor	NCT03015701	III	Meningioma	Completed
			NCT01419535	I/II	Glucose intolerance	Completed
			NCT00255177	II	Hepatitis C	Completed
			NCT00140478	II	Androgen independent prostate cancer	Completed
			NCT03015701	III	Meningioma	Completed
NAC			NCT00775476	II	Systemic lupus Erythematosus	Recruiting
			NCT03306979	II	Vascular impairment	Completed
			NCT05241262	I	m.3243A>G mutation	Recruiting
			NCT05287724	Early phase-I	Prurogenic stimuli	Completed
			NCT04481048	II	Neurofibromatosis type 1	Active, not recruiting
			NCT05122559	II	Progressive multiple sclerosis	Recruiting

## Future directions and challenges in ferroptosis-based glioblastoma therapy

### Biomarker development for clinical implementation

A critical challenge in clinically applying ferroptosis-inducing therapies lies in identifying reliable biomarkers for tumor susceptibility. This difficulty stems from the heterogeneous expression of ferroptosis-related genes across different glioblastoma subtypes. Prospective validation of a 12-gene ferroptosis susceptibility signature has shown 82% accuracy in predicting response to ferroptosis inducers [177]. Furthermore, emerging non-invasive ferroptosis-specific PET tracers (e.g., ^68^Ga-ferroptosis probe), have demonstrated promise in preclinical models with 89% tumor specificity [178]. Developing and validating such biomarkers is therefore essential for effective patient stratification in future clinical trials.

### Advanced delivery systems for brain penetration

The BBB remains a formidable obstacle to delivering therapeutic agents to brain tumors. Consequently, current ferroptosis inducers achieve only limited tumor penetration. To overcome this challenge, novel iron-chelating nanoparticle platforms can be used for the delivery of ferroptosis inducers. These nanoparticles have shown 8-fold greater accumulation in orthotopic glioblastoma models compared to free drugs [179]. Furthermore, focused ultrasound-mediated BBB opening can be used, which enhance drug delivery efficiency by 76% [180]. Convection-enhanced delivery of liposomes has also increased drug distribution volume by 3.2-fold in primate models [181]. Effectively coupling these technological advancements with ferroptosis inducers is essential to significantly enhance the efficacy of glioblastoma treatment.

### Tumor heterogeneity and resistance

Future studies should investigate the therapeutic potential of targeting ferroptosis resistant subpopulations in glioblastoma, which comprise 12–28% of tumor cells as identified by single-cell multi-omics profiling [182]. Given the upregulation of compensatory metabolic pathways, including FSP1 and DHODH, in resistant glioblastoma cells, combinatorial inhibition of GPX4 and FSP1 represents a promising therapeutic avenue that warrants further investigation.

### Tumor recurrence dynamics

Future research should focus on targeting ferroptosis resistant subclones that drive glioblastoma recurrence, as identified by multi-omics profiling in 89% of recurrent tumors. Key resistance mechanisms such as Nrf2 activation, enhanced trans-sulfuration, and improved DNA repair may be overcome through dual GPX4/Nrf2 inhibition, metabolic blockade, and HDAC-based epigenetic modulation. Development of predictive biomarkers, combination therapies preserving T-cell function, and microenvironmental interventions like xCT knockout offer promising avenues. Future clinical trials integrating GPX4 inhibitors with radiotherapy may define therapeutic windows that disrupt recurrence-driving adaptations and reshape glioblastoma treatment paradigms.

### Immune system interactions and therapeutic optimization

Future studies should refine ferroptosis-based immunotherapy by addressing the adaptive immunosuppression that emerges with prolonged treatment in glioblastoma. Clinical data show that sustained ferroptosis induces CD8⁺ T-cell exhaustion, MDSC expansion, and antigen presentation loss. To overcome this, preclinical models support intermittent dosing, STING agonists to enhance DC cross-priming, and CSF-1R inhibition to block myeloid-driven immunosuppression while preserving ferroptotic efficacy. Future trials should evaluate sequential ferroptosis-immunotherapy regimens may reveal strategies to elicit durable, tumor-specific immune responses and improve glioblastoma outcomes.

### Metabolic crosstalk in the peritumoral niche

Disrupting metabolic crosstalk within the glioblastoma peritumoral niche represents a promising frontier in enhancing ferroptosis efficacy. Future research should prioritize strategies that target the tumor-supportive roles of astrocytes, microglia, and neurons, which collectively maintain high cysteine availability and GSH synthesis in resistant tumors. Approaches such as astrocyte-specific xCT knockout, glutamate release inhibitors, and multifunctional nanocarriers co-delivering ferroptosis inducers with metabolic blockers may demonstrate success in reducing cysteine supply and boosting tumor susceptibility to ferroptosis. As these strategies move into early-phase clinical trials, efforts to optimize delivery systems and ensure neural safety will be critical to translating microenvironment-targeted ferroptosis therapies into effective treatments for resistant glioblastoma.

### Emerging therapeutic frontiers and translation challenges

Emerging innovations are poised to advance ferroptosis-based therapies for glioblastoma, including nanoparticle vaccines carrying lipid peroxidation products that elicit strong immune memory and achieve complete responses in preclinical models. Chronotherapy, guided by circadian regulation of iron metabolism, may optimize treatment timing and efficacy, while next-generation PET tracers offer precise, early detection of therapeutic response. However, translating these strategies requires overcoming key challenges such as standardizing ferroptosis monitoring assays, addressing sex-based differences in sensitivity, integrating with modalities like tumor-treating fields, and ensuring safety in long-term iron modulation. Coordinated global efforts and dedicated clinical trial networks will be crucial to accelerate clinical translation and improve glioblastoma patient outcomes.

### Safety considerations and normal tissue protection

As ferroptosis emerges as a promising therapeutic strategy in glioblastoma, future efforts must carefully address associated toxicities. Clinical data indicate that high ferroptosis susceptibility may increase the risk of peritumoral edema, underscoring the need for protective strategies. Preclinical findings suggest that selective astrocyte protection via Nrf2 activation can significantly reduce off-target toxicity without compromising tumor cell death. Advancing tumor-selective ferroptosis inducers with favorable safety profiles will be essential for translating this approach into clinically viable treatments.

## Conclusion

Ferroptosis represents a transformative therapeutic opportunity in glioblastoma treatment, offering a novel mechanism to overcome the limitations of conventional therapies. By targeting iron-dependent lipid peroxidation, ferroptosis induction bypasses key resistance pathways associated with chemotherapy, radiotherapy, and immunotherapy, while exploiting glioblastoma’s metabolic vulnerabilities. The integration of ferroptosis inducers with standard therapies has demonstrated significant potential to enhance treatment efficacy evidenced by improved survival in preclinical models and early clinical trials, particularly in overcoming MGMT-mediated chemoresistance, radio-resistance, and immunosuppression. However, challenges such as tumor heterogeneity, adaptive resistance mechanisms, and the need for precise delivery systems must be addressed to ensure durable clinical benefits. Future research should focus on refining ferroptosis-targeted strategies through optimized combination approaches, biomarker-driven patient selection, and advanced drug delivery technologies. Collaborative efforts across basic, translational, and clinical research will be essential to fully harness the potential of ferroptosis as a cornerstone of next-generation glioblastoma therapy, ultimately improving outcomes for patients facing this devastating disease.
